# Is tension band wiring technique the "gold standard" for the treatment of olecranon fractures? A long term functional outcome study

**DOI:** 10.1186/1749-799X-3-9

**Published:** 2008-02-22

**Authors:** Byron E Chalidis, Nick C Sachinis, Efthimios P Samoladas, Christos G Dimitriou, John D Pournaras

**Affiliations:** 1 1^st ^Orthopaedic Department of Aristotle University of Thessaloniki, Greece

## Abstract

**Background:**

Tension band wiring (TBW) remains the most common operative technique for the internal fixation of olecranon fractures despite the potential occurrence of subjective complaints due to subcutaneous position of the hardware. Aim of this long term retrospective study was to evaluate the elbow function and the patient-rated outcome after TBW fixation of olecranon fractures.

**Methods:**

We reviewed 62 patients (33 men and 29 women) with an average age of 48.6 years (range, 18–85 years) who underwent TBW osteosynthesis for isolated olecranon fractures. All patients were assessed both clinically with measurement of flexion-extension and pronation-supination arcs and radiologically with elbow X-Rays. Functional outcome was estimated using the Mayo Elbow Performance Score (MEPS), Visual Analogue Scale (VAS) subjective pain score and VAS patient satisfaction score. Follow up: 6–13 years (average 8.2 years).

**Results:**

There was a higher prevalence of fractures among men until the 5th decade of life and among women in elderly (p = 0.032). Slip or simple fall onto the arm was the main mechanism of injury for 38 fractures (61.3%) while high energy trauma, such as fall from a height (> 2 m) or road accident, was reported in 24 fractures (38.7%). Hardware removal performed in 51 patients (82.3%) but 34 of them (66.6% of removals) were still complaining for mild pain during daily activities. The incidence of pin migration and loosening was not statistically decreased when penetration of the anterior ulnar cortex was accomplished (p = 0.304). Supination was more often affected than pronation (p = 0.027). According to MEPS, 53 patients (85.5%) had a good to excellent result, 6 (9.7%) fair and 3 (4.8%) poor result. The average satisfaction rating was 9.3 out of 10 (range, 6–10) with 31 patients (50%) to remain completely satisfied from the final result. Degenerative changes recorded in 30 elbows (48.4%). However, no correlation could be found between radiographic findings and MEPS (p = 0.073).

**Conclusion:**

Tension band wiring fixation remains the "gold standard" for the treatment of displaced and minimally comminuted olecranon fractures. In long term, low levels of pain may be evident regardless of whether the metalware is removed and degenerative changes have been developed.

## Background

Olecranon fractures are common injuries of the proximal ulna which constitute about 10% of all upper extremity lesions [1]. The fractures are usually isolated but associated lesions can be occurred in complex injuries and polytrauma cases [2,3]. Due to the intra-articular extension of fractures, anatomic reduction and early mobilization should be achieved in any case [4]. It is known that only undisplaced fractures (5% of total) are treated conservatively while displaced fractures (95% of total) are submitted to operative treatment [4-6].

Tension band wiring (TBW) which was introduced by Weber and Vasey [7] remains the most widespread method for fracture osteosynthesis [8-10]. However, a number of complications such as infection, non-union, malunion and ulnar nerve palsy could compromise the effect of operative treatment in up to 10% of cases [11-13]. Moreover, the subcutaneous placement of K-wires and their potential migration may be responsible for local pain, secondary displacement and wound healing problems [14].

The purpose of this study was to determine the clinical and radiological outcome after tension band wiring of olecranon fractures and to record the incidence of hardware removal and residual pain or disability.

## Methods

Between 1993 and 2000, 103 patients – from a rough total of 74200 admitted cases to Orthopaedic Emergency Department – presented themselves with an olecranon fracture (overall incidence rate 0.0014%). Isolated fractures without severe concomitant injuries or complex lesions of the affected elbow were recognized in 89 patients and 77 of them were treated with TBW technique. Two patients died, 7 patients were lost despite exhaustive search processes and 6 patients refused examination for different reasons. The remanining 62 patients were identified and formed the study group.

**Figure 1:**
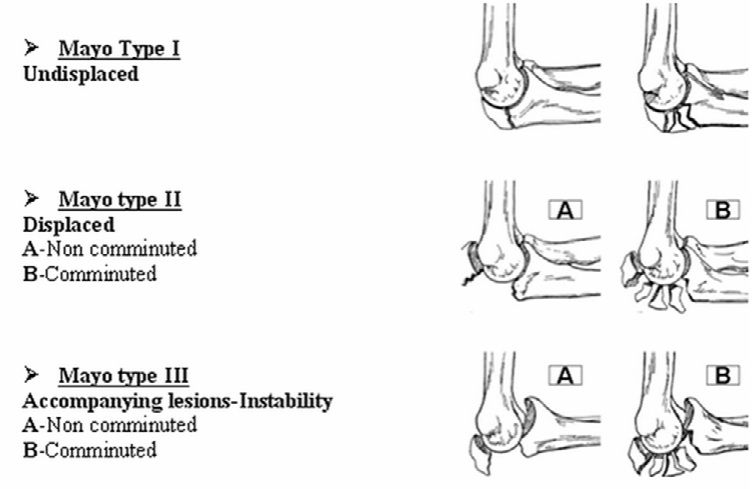
**Mayo Classification for olecranon fractures**. Adapted from [4].

The fracture pattern was assessed using the Mayo classification [4] which takes into account the degree of fracture displacement and comminution as well as the stability of the elbow joint (Figure 1). The surgical procedures were carried out with the patient in a supine or lateral decubitus position under general or regional anesthesia. A tourniquet was inflated and the fracture site was approached via a posterior midline skin incision. In each case, the ulnar nerve was identified with palpation but neither its release nor its transposition was primarly performed. Fracture osteosynthesis was achieved with the insertion of two parallel 1.8 mm Kirschner wires from the tip of the olecranon and a 18 gauge wire in a figure-of eight fashion. Major intraoperative goal was the perforation of the ulnar anterior cortex in an effort to increase fixation stability and to minimize pin migration. The proximal end of K-wires was bent and the cerclage wire was placed through a predrilled transverse hole in the distal fragment and under the triceps tendon. Subsequently, the cerclage wire was tightened to create interfragmentary compression. One or two-knot technique of tightening was utilized according to surgeon's preference. The entire process was done under fluoroscopic guidance. Intraoperative result was considered acceptable when less than 2 mm intra-articular gap or displacement was apparent.

The postoperative protocol included antibiotics administration (cefuroxime) for 24 hours and progressive mobilization of the elbow joint. In cases of fracture comminution (Types IIB and IIIB) a posterior splint with the elbow in a semiflexed position was applied for a period of 2–3 weeks with the aim to prevent fracture collapse and displacement. Anteroposterior and lateral elbow radiographs were repeated at regular intervals until evidence of union was detected. Finally, all the patients were recalled to attend a specially set up clinic for the final assessment with respect to the purpose of the study. Ethics Committee approval was obtained. Flexion-extension arc of the elbow and pronation-supination arc of the forearm were measured with a goniometer. Patient-rated outcomes evaluated with the Mayo Elbow Performance Score (MEPS), Visual Analogue Scale (VAS) subjective pain score (10 = unbearable pain) and VAS patient satisfaction score (10 = complete satisfaction) [2]. Degenerative changes were described as the presence of at least one of the following radiological signs: subchondral cysts, subchondral sclerosis or osteophytes (separate or together) [8]. The duration of follow up was from 6 to13 years (average 8.2 years).

### Statistics

The microsoft excel program was used for the creation of the graphs and the SPSS program 12.0 (SPSS Inc, Chicago, IL, USA) for the creation of statistics. Data analysis was conducted with Chi-square test and Student's t-test. P values less than 0.05 were considered to be statistically significant.

## Results

**Figure 2:**
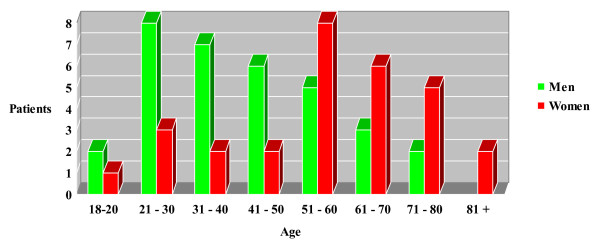
Distribution of olecranon fractures according to sex and age.

From the total of 62 patients who were included in the study, there were 33 men and 29 women with an average age of 48.6 years (range, 18–85 years). The frequency of fractures was higher in men until the 5th decade of life but altered in older decades towards women (p = 0.032) (Figure 2). The left elbow was affected in 35 patients (56.4%) and the right in 27 patients (43.6%). Regarding the mechanism of injury, slip or simple fall onto the arm were responsible for 38 fractures (61.3%). In the remaining cases, the fractures were a result of a high energy trauma, such as fall from a height (> 2 m) (15 cases, 24.2%) or road accident (9 cases, 14.5%).

According to Mayo classification for olecranon fractures, the distribution of lesions was as follows: 40 Type IIA (64.5%), 13 Type IIB (21%), 8 Type IIIA (12.9%) and 1 Type IIIB (1.6%). The average period of hospitalization was 3.7 days (median = 2; min = 1, max = 10).

Wound infection developed in 4 patients (6.5%). In 2 cases intravenous antibiotics proved adequate for the eradication of the infection while in the other 2 elbows wound irrigation and surgical debridement were performed.

Reduction was maintained in all elbows until fracture union. No malunions or ulnar nerve palsies complicated the postoperative period. However, non-union was encountered in 2 patients (3.2%) after high velocity injuries who had Type IIB and IIIB fractures accordingly. Re-osteosynthesis in combination with iliac bone graft led to uneventful healing.

**Figure 3:**
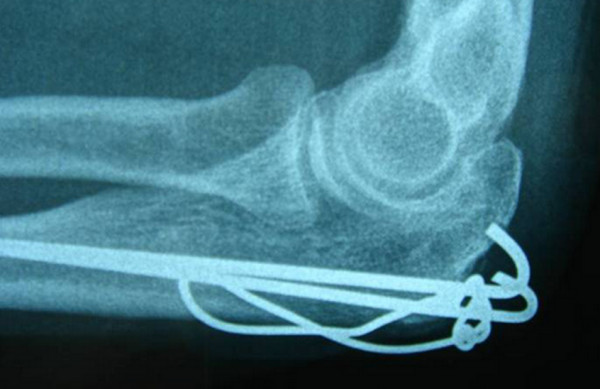
**Pin migration**. Lateral radiograph of the right elbow 2 years after TBW of an isolated olecranon fracture in a 42-year-old woman. Despite fracture union, backing out of K-wires was evident. The patient was complaining for pain during elbow movements (VAS pain subjective score = 4) and skin irritation. Removal of metalwork was followed by partial resolution of symptoms as mild discomfort was reported even 8 years postopeartively (VAS pain subjective score = 2).

**Figure 4:**
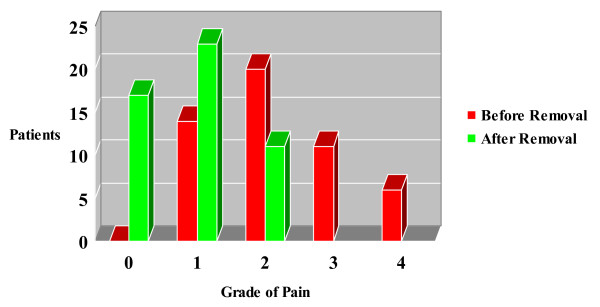
Visual Analogue Scale (VAS) subjective pain score in patients before and after hardware removal.

The anterior ulnar cortex was perforated by both K-wires (group A) in 39 fractures (62.9%), by one (group B) in 5 fractures (8.1%) and by none (group C) in 18 fractures (29%). Hardware removal recorded in 51 patients (82.3%) due to pin prominence, localized pain or direct complaint defined as the patient being "bothered" by the metalware (VAS 1–4). The above event was not significantly affected by pin position as it was found with a frequency of 76.9% (30 fractures) in group A, 100% in group B (5 fractures) and 88.8% in group C (16 fractures) (p = 0.304). It is noteworthy that after metalwork removal, 34 from the 51 patients (66.6% of removals) were still complaining for mild pain (VAS 1–2) (Figures 3 and 4).

Only 2 patients (3.2%) had a significanly reduced flexion arc -50 and 45 degrees respectively- affecting the functional outcome. The rest of cases had some degrees of flexion or extension deficit without being functionally disabled. Supination was more often affected than pronation (p = 0.027) (Table 1).

**Table 1:** Elbow range of motion (ROM) in affected and unaffected limb.

**Elbow**	**Flexion**	**Supination**	**Pronation**
**Affected**	136.5 ± 7.9*	73.6 ± 4**	74.3 ± 3.2
**Unaffected**	141.4 ± 1	82.4 ± 1.5	76,6 ± 2.1

± = Std. Deviation, *(p = 0.031),** (p = 0.027).

**Figure 5:**
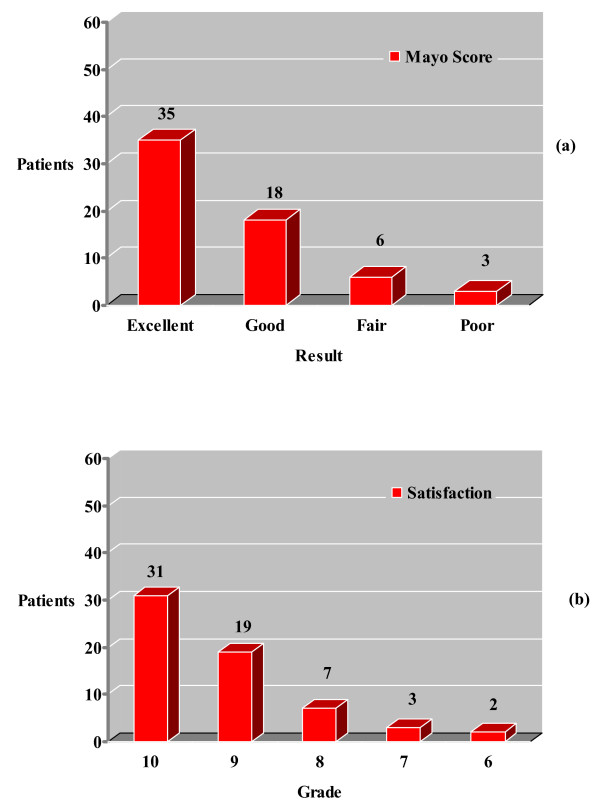
Mayo Elbow Performance score (MEPS) (a) and Visual Analogue Scale (VAS) patient satisfaction score (b).

**Figure 6:**
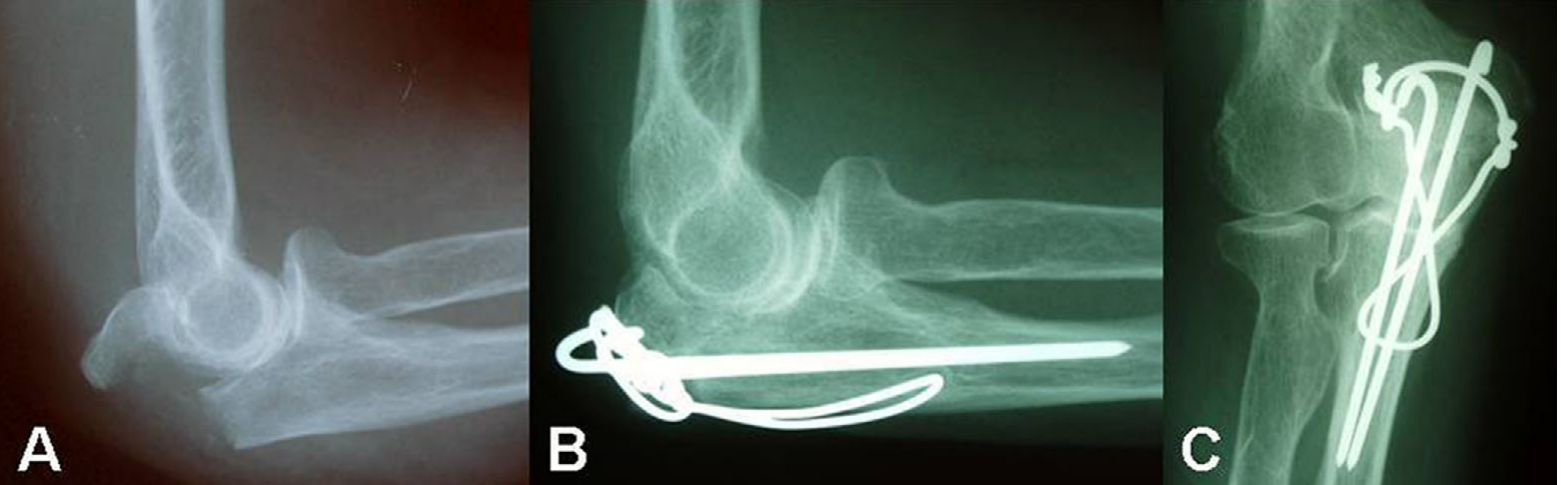
**Elbow degeneration and olecranon fracture**. Mayo Type IIA fracture of the left olecranon after a fall in a 52-year-old woman **(A)**. Lateral **(B) **and anteroposterior radiographs **(C) **at 7 years postoperatively showed signs of subchondral sclerosis and osteophytes formation in radioulnar and ulnohumeral joints.

Using the MEPS, 53 patients (85.5%) had a good to excellent result, 6 (9.7%) fair and 3 (4.8%) poor result. The average satisfaction rating was 9.3 out of 10 (range, 6–10) with 31 patients (50%) to state complete satisfaction of the final result (Figure 5). Degenerative changes were found in 30 elbows (48.4%) but no correlation with the MEPS was identified (p = 0.073) (Figure 6).

Finally, 56 out of 62 patients (90.3%) returned to their original work and no reduction of the level of athletic activitiy was evident among the 16 patients (25.8%) who reported some kind of involvement in sports.

## Discussion

Olecranon fractures may be caused by direct injury to the posterior part of the elbow joint or indirectly by forces generated within the triceps muscle during a fall on a partially flexed elbow [15]. The clinical picture is obvious and conventional radiographs are usually sufficient to depict the lesion and the potential associated injuries [4].

In the herein study, the incidence of olecranon fractures showed a higher prevalence among men until the 5th decade of life and among women in older ages. Similarly, Rommens et al [9] reported that nearly half of men with olecranon fractures were between 21 to 40 years of age and 40% of women between 61 and 80 years old. Regarding the side of injury, 56.4% of fractures in our series were located in the left limb while Akman et al [16] observed a predominance of right elbow in 60% of cases.

Tension band wiring (TBW) technique, which is relied on the principle of converting posterior tensile forces to articular compressive forces, has gained widespread acceptance for the surgical treatment of olecranon fractures [17-19]. Many authors have suggested various modifications in order to improve the biomechanical properties of the technique [20]. Rowland and Burkhart [21] gave an emphasis on the mathematical need to put the transverse hole for the figure-of-eight tension band wire anterior to the intramedullary pin. The above hypothesis wasn't confirmed by Paremain et al [22] as the results of their biomechanical study indicated no significant differences in yield loads or stiffness values between the Rowland-Murkhart and AO tension band wiring techniques.

In spite of the efficacy of TBW fixation even in cases with severe fracture displacement and comminution, many patients express pain or discomfort due to subcutaneous position of the K-wires and the relevant incidence of metalware removal may be raised to 87% [8-10]. Rommens et al stated that suboptimal pins placement (K-wires which are not inserted parallel or they do not transverse the opposite cortex of the proximal ulna) was not correlated with increased rate of implant loosening or secondary procedures [9]. As the above finding was also evident in our study, we advocate that insertion of K-wires into the anterior ulnar cortex may increase TBW construct stability and stiffness but it couldn't prevent posterior pin migration when active motion of the elbow joint has beeen commenced. Furhermore, hardware removal seems not always to be a panacea for symptoms resolution as 66.6% of TBW removals were still complaining for mild pain or discomfort. Romero et al [13] noted that backing-out of K-wires and metalwork prominence could not justify alone the need for TBW removal and they should not be considered entirely responsible for patients' subjective complaints.

To avoid hardware problems with TBW technique, some authors have recommended plating osteosynthesis for fracture stabilization [23,24]. Bailey et al [2] reported high patient satisfaction (9.7/10) with a low pain rating (1/10) after plate fixation in Mayo types II and III fractures. Although plate removal was performed in 20% of cases the mean DASH score was consistent with almost normal upper extremity function. Hume and Wiss [25], in a prospective randomized study, found that the application of plates and screws in comparison with TBW construct demonstrated less frequent loss of reduction and better clinical and radiographic results. During the last decade the policy in our department is to use plate fixation when fracture comminution (Types IIB and IIIB) couldn't support compression with the TBW technique. Current low profile, precontoured titanium olecranon plates fit anatomically to the bone, cause less soft tissue irritation, increase fracture stability and allow immediate mobilization of the elbow joint.

Various degrees of postoperative elbow stiffness and deficit of range of motion have been reported in literature after surgical treatment of olecranon fractures [1,26,27]. Ring et al [28] and Teasdall et al [29] reported that patient compliance, fracture comminution and extension into the ulnar diaphysis or coronoid process, concomitant radial head fracture and elbow instability may lead to inferior results. On the other hand, Villanueva et al [10] noted that fracture comminution does not necessary have a harmful effect on both clinical and radiological outcome.

Degenerative changes are not uncommon after olecranon fractures and they have been related to the length of follow up [10]. Karlsson et al [8] found that with a mean of 19 years after isolated olecranon fractures 50% of the patients developed degenerative changes. However, these patients did not report any substantial symptoms and no correlation could be found between radiographic findings and patient subjective outcome. In 48.4% of our patients degenerative changes were identified after an average of 8.2 years postoperatively. The main point is that the functional scores of patients with degenerative changes weren't different of those with normal X-rays. Proper studies and further investigation are required to address the clinical importance of the above issue.

Non-union, ulnar nerve palsy and wound infection have been described in approximately 2–10% of olecranon fractures [4,27]. Even though the subcutaneous position of the Kirschner wires and their subsequent migration may be responsible for secondary displacement and wound healing problems, careful operative technique and appropriate soft tissue management are of greatest importance in order to minimize the aforementioned complications.

## Conclusion

Tension band wiring fixation for isolated olecranon fractures leads to good elbow function and minimal loss of physical capacity. The technique remains the "gold standard" for the treatment of displaced and minimally comminuted olecranon fractures despite the introduction of new implants designed specifically to address the problems of wound irritation and metalware removal. In long term, low levels of pain and elbow degenerative changes may be evident but no clear correlation could be established between radiological and clinical result.

## Competing interests

The author(s) declare there are no competing intersets.

## Authors' contributions

BC wrote the paper. NS and ES collected and statistically analyzed the data. CD and JP conceived, designed and revised the study. Each author read and approved the final manuscript.
